# Using the ICF Framework to Assess Communicative Competence in Dyadic Communication among Children and Adolescents Who Use Augmentative and Alternative Communication Devices in Taiwan

**DOI:** 10.3390/bs12110467

**Published:** 2022-11-21

**Authors:** Meng-Ju Tsai

**Affiliations:** 1 Department of Speech-Language Pathology and Audiology, Chung Shan Medical University, No. 110: Sec. 1, Chien-Kuo North Road, Taichung City 402, Taiwan; mjtsai@csmu.edu.tw; 2 Speech and Language Therapy Room, Chung Shan Medical University Hospital, Taichung City 402, Taiwan

**Keywords:** augmentative and alternative communication (AAC), international classification of functioning, disability and health (ICF), communicative competence

## Abstract

Augmentative and alternative communication (AAC) devices enable children and adolescents (CAD) with communication disorders to communicate competently and develop friendships through communicative competence (CC). Existing assessment tools are unable to indicate whether CAD aged 0 to 18 years would competently use the subsidized AAC devices provided by the Ministry of Health and Welfare in Taiwan. This study, thus, aimed to develop an assessment tool by using the International Classification of Functioning, Disability and Health (ICF) to measure CC in dyadic communication among CAD using AAC devices. Five speech-language pathologists (SLPs), five special education teachers, and four AAC experts (14 in total) selected codes relevant to the four domains of CC via the Delphi method. Next, they categorized the selected codes into one of the four domains of CC through a face-to-face expert panel. A total of 112 codes were listed in the tool and fully classified into the four domains of CC. Among these, seven codes were concurrently placed under two domains of CC. Consequently, this study developed an assessment tool by employing the ICF for children and youth core set using universal qualifiers to measure the relative levels of CC in dyadic communication among CAD who use AAC devices in their daily life.

## 1. Introduction

### 1.1. Augmentative and Alternative Communication (AAC) Devices in Taiwan

Taiwan, with a population of about 23.2 million people, is classified as a developed nation; 66.3% of Taiwanese people over six years of age speak Mandarin, while 31.7% speak the Taiwanese dialect [1]. An estimated 52,142 children and adolescents (CAD) in the country live with disabilities, and about 43,156 of those aged 0 to 18 years were living with intellectual and/or developmental disabilities (IDD) in 2021 [2]. CAD with complex communication needs (CCN) secondary to IDD frequently benefit from augmentative and alternative communication (AAC), including high-tech (e.g., speech-generating devices [SGDs]) and low-tech (e.g., picture boards) AAC devices [3]. The MOHW subsidizes the purchase of AAC devices, which are assessed and recommended by Type B assistive technology evaluators (i.e., licensed speech-language pathologists, or SLPs) [4]. Although Assistive Device Evaluation Report #12, proposed by the Social and Family Affairs Administration [SFAA] [5] of MOHW, is used for assessment and subsidies; this assessment tool is not specific to CAD. Furthermore, it cannot indicate whether CAD aged 0 to 18 years would competently use the subsidized AAC devices in their daily communication life [6]. Without follow-up assessment tools, these subsidized AAC devices might be abandoned [6].

### 1.2. The Current Assessment of AAC Devices

The ultimate goal of introducing CAD to AAC devices is to ensure they communicate competently while using said devices [7]. Communicative competence (CC) facilitates the realization of an individual’s fundamental needs, rights, and power of communication [8]. CAD also develop friendships through CC [9]. CC, as proposed by Light [10], contains four domains: (a) linguistic, (b) operational, (c) social, and (d) strategic. Linguistic competence refers to skills in one’s native language spoken verbally in the community, as well as the symbols used on AAC devices that are learned, but not innately understood [11]. One’s native language continues to be a primary input (i.e., understanding), while symbols are a primary output (i.e., expression) throughout one’s life. Operational competence encompasses the technical skills needed to effectively implement motor control and use AAC devices, including access methods (e.g., direct selection among a large set of messages and sequentially programmed timed selection for messages) and the operation of the device’s features (e.g., switches, volume control, coding systems, and output mode selection) [10,11]. Light, Beukelman and Reichle [11] argued that these skills require motivation (e.g., trusting AAC devices), attention (e.g., attention to tasks requiring corresponding skills), and/or cognition (e.g., understanding the required motor planning steps).

Social competence involves using social communication and pragmatic skills to meet communication goals. It also includes interpersonal aspects of communication [3]. The pragmatic skills entail initiating topics for conversation, repairing communication breakdowns, exchanging communication turns (i.e., turn-taking), and terminating communication [3,11,12]. The interpersonal aspects of communication include demonstrating an interest in one’s communication partners (CPs), actively participating in communication, being responsive to others, putting CPs at ease, and developing a positive rapport with others [11,13]. Finally, strategic competence encompasses a collection of adaptive skills that are learned to compensate for internal restrictions (e.g., linguistic, operational, and/or social) and/or external restrictions (e.g., CPs’ constraints and/or the slow rate of communication when using an AAC device) [10,11,14]. For example, a speech-generated message (e.g., *no*) from an AAC device might be used strategically to signal that a CP’s message cannot be understood. All CPs need different adaptive skills over time in their daily communication life [11]. In addition, the above mentioned four domains are interrelated [8,10]; that is, the use of words, knowledge, judgment, and skills when using an AAC device concurrently involves more than one domain of CC [15]. For instance, producing a speech-generated message (e.g., *help*) from an AAC device to call someone’s attention and then directly pointing to a color photo on the device to request a cup of water requires using all four domains simultaneously. The steps needed to achieve the above mentioned action include memorizing the color photos and the symbols they express (i.e., linguistic competence); directly pointing to a color photo to make a request (i.e., operational competence); initiating the request and actively communicating (i.e., social competence); and making an initial sound to draw the CP’s attention to oneself (i.e., strategic competence).

CC is dyadic, learned, co-constructed, relative, and performance-based [8,16]. First, CC is grounded in an interpersonal dyad (i.e., two CPs) or a group, rather than the individual [14,16,17,18]. Each CP in the dyad alternately acts as the listener or speaker [19]. CC develops through cooperation as a result of social interactions in communication dyads [20]. Second, CC is learned [16]. Dyadic CPs (e.g., a person who uses an AAC device and a CP) must learn the appropriate communication repertoire, including when (and when not) to talk and what to talk about with whom, when, where, and in what manner [21]. Light [10] stressed that people who use AAC devices have to learn linguistic, operational, social, and strategic skills to “speak” through their devices, and CPs have to learn diverse skills (e.g., filling communication gaps) to communicate with those who use AAC devices [18]. Third, CC is co-constructed by CPs. It thereby actively facilitates communication for people who use AAC devices and often passively participate in dyadic communication [16,22,23]. Fourth, CC is relative and dynamic [16]; it is not absolute or static [14], but rather varies from high to low levels [18]. Perceptions of the levels of CC do not need criteria and norms [14,18], and fluctuate according to diverse communication contexts (e.g., discussing food) and dyads (e.g., participants A1 and A2) [16]. Fifth, CC is measured based on performance [16]. CC is gauged during real-life communication [16], rather than in a standardized or uniform setting [24]. Hence, CC should be measured among communicators (i.e., CAD who use AAC devices), CPs (i.e., people who communicate with CAD), and others (e.g., family members, SLPs, and special education teachers) [18,25].

Currently, there are four assessment tools for measuring CC that have been assessed in extant studies and in actual practice (please refer to Supplementary Materials for details), including the Communicative Competence Rating Scale (CCRS) [26], the Communicative Competence Scale (CCS) [27], the Communication Supports Inventory-Children and Youth (CSI-CY) [28,29], and the Dynamic AAC Goals Grid 2 (DAGG-2) [30]. However, considering the four domains and perceptions of CC (e.g., dyadic, co-constructed), each of the above-mentioned tools has limitations. First, the CCRS, CCS, and DAGG-2 do not reflect the perceptions of CC, which imply a dyadic, co-constructed characteristic. That is, these tools focus on children and/or adolescents only, and fail to consider dyadic CPs. Second, the CCRS, CCS, and CSI-CY were normed on 11–18-year-olds, 12–20-year-olds, and 5–20-year-olds, respectively, but not on 0–4-year-olds. Furthermore, the DAGG-2 does not specifically address CAD, but rather people of various ages. Third, the CCRS, CCS, CSI-CY, and DAGG-2 have been judged only by CPs and/or healthcare providers, without any self-assessment from the communicators themselves (i.e., CAD who use AAC devices). Fourth, the CCRS and CCS have been developed for specific research purposes (research applications) and may be unfamiliar to global, multidisciplinary practitioners and scholars. Moreover, definitions for each point of the Likert scale (i.e., strongly disagree; disagree; neither agree nor disagree; agree; and strongly agree) for each listed assessment item are lacking. Fifth, some domains of CC are not included in the CCS, which loosely addresses strategic and social competence but does not deal with linguistic and operational competence. Moreover, the four domains and the nature of CC are not reflected in these current assessment tools. In sum, existing tools for measuring CC cannot determine whether 0–18-year-old CAD would competently use the subsidized AAC devices in their daily communication life in Taiwan. Therefore, a universally accepted and standardized tool for determining the potential benefits of subsidized AAC devices for CAD in Taiwan is needed for social and educational services.

### 1.3. The International Classification of Functioning, Disability, and Health (ICF)

The International Classification of Functioning, Disability and Health (ICF) and the International Classification of Functioning, Disability and Health for Children and Youth (ICF-CY), officially endorsed by the World Health Organization (WHO), use a universal, standardized language for clinical, public health, and research applications [31,32]. The ICF and ICF-CY document the functioning of individuals by considering the interactions between health conditions and contextual factors, and allow for consistent communication about health and health care across the world in various disciplines and sciences [31,32]. The ICF-CY documents bodily functions and structures, activity limitations, participation restrictions, and environmental factors emerging during infancy, childhood, and adolescence [31]. Despite limited knowledge in applying the ICF-CY among CAD who use AAC devices [33], several scholars [12,34] have suggested that the ICF-CY is very appropriate to be applied to CAD. The ICF-CY can be used by service providers, consumers, and others concerned with the health, education, and well-being of CAD [31], especially for describing communication and social participation [35]. Four components—*Body Functions (b), Body Structures (s), Activities and Participation (d)*, and *Environmental Factors (e)*—are included in the ICF-CY. The physiological functions of body parts are encompassed by *Body Functions (b)*, while anatomical body parts such as organs, limbs, and their components are subsumed under *Body Structures (s)* [31]. The interactions of CAD in their families, schools, and communities are focused on *Activities and Participation (d)* [36], while the physical, social, and attitudinal environments external to CAD are *Environmental Factors (e)* [31]. However, personal factors (e.g., personal attributes) are not classified due to their associations with extensive social and cultural variance [31].

The ICF-CY contains 1,645 alphanumeric codes (e.g., *d132*) [31,34,37] that are hierarchically organized at the first, second, third, and fourth levels of classification; they are mutually exclusive (i.e., not sharing the same attributes) [31,34]. There are 522 codes in *Body Functions (b)*, 321 in *Body Structures (s)*, 543 in *Activities and Participation (d)*, and 259 in *Environmental Factors (e)* [31]. Five levels of universal qualifiers—(0) no impairment, difficulty or barrier to (4) complete impairment, difficulty or barrier—in the ICF-CY are used to document the severity or magnitude of the body’s functions and structures, activity limitations, participation restrictions, and environmental factors using direct measurements, observation, first-hand interviews, and/or professional judgment [31].

To improve coverage of the transitions across the lifespan, and for easier maintenance and reduced redundancy of work, the World Health Organization [WHO] [38] announced the merger of the ICF-CY and the ICF, and that the codes in the ICF-CY will be added to the ICF. These codes in the ICF-CY were maintained as a specially derived classification and linearization of the ICF, but no further updates were made. Scholars, e.g., [39,40] concluded that CC can be measured through the ICF-CY, which offers a valid and reliable framework. First, potential assessment items can be converted from the universal alphanumeric codes (e.g., *d132*) [41], and assessment items can be standardized from the standardized language [37]. Second, the four domains of CC that determine the functioning of CAD interact better with the four components of the ICF-CY rather than with a traditional focus on single outcomes [28,42]. Researchers e.g., [24,28,36,41] have concluded that *Activities and Participation (d)* and *Environmental Factors (e)*—which contain codes related to communication, with a focus on the interactions of CAD with their families, schools, and communities—can better capture the performance of CC in an interpersonal dyad. Third, the perceptions of CC (e.g., dyadic, co-constructed) meet the frameworks of the ICF-CY [8,16]. *Activities and Participation (d)*, a category focused on the interactions of CAD in their families, schools, and communities, and *Environmental Factors (e)*, centered on the physical, social, and attitudinal environments external to CAD, consider the interactions (e.g., co-construction) of both CAD and others (e.g., CPs) in daily communication life not solely CAD. In addition, the five levels of universal qualifiers, which document activity limitations, participation restrictions, and environmental factors, are relative and dynamic (as opposed to absolute and static). The documentation—performed via direct measurement, observation, first-hand interviews, and/or professional judgment—derived from the ICF-CY is similar to the CC exhibited by CAD, CPs (e.g., family members), and healthcare providers (e.g., SLPs). Simeonsson, Björck-Åkessön and Lollar [36] further established that the CC of people in families, schools, and communities can be understood through the ICF-CY.

However, administering these codes limits the use of the ICF-CY in specific healthcare contexts and daily practices [37,43]. Simeonsson, et al. [44] and Pan, et al. [45] indicated that obtaining a certain minimum number of codes is necessary for numerous groups and settings. A core set is a selected set of codes that are relevant to most people with a certain health condition or in a particular healthcare context; the codes comprehensively portray people’s level of function [46,47,48,49]. A core set is an evidence-based shortlist, including a brief core set (20–30 codes) and a comprehensive core set (70–100 codes) [37,50]. The core set allows global, multidisciplinary practitioners and scholars to jointly establish minimum standards for measuring functioning and health using clinical and multi-professional assessments while considering environmental factors [34,43,48]. Bernabeu, Laxe, Lopez, Stucki, Ward, Barnes, Kostanjsek, Reed, Tate, Whyte, Zasler and Cieza [34] and Pan, Hwang, Simeonsson, Lu and Liao [45] found that the core set can, over time, quantify the severity of impairments in *Body Functions (b)* and *Body Structures (s)*, the limitations of *Activities and Participation (d)*, and *Environmental Factors (e)* or barriers experienced by diverse kinds of people with communication disorders. The development of a core set can be used to assess, intervene, and gauge people’s functioning for multiple disciplines [34,50,51].

Unfortunately, scant research has critically examined the ICF-CY core sets to measure CC among CAD aged 0 to 18 years who use AAC devices [52,53]. Hence, the current study aimed to develop an assessment tool by using the ICF framework to measure CC in dyadic communication among CAD who use AAC devices in Taiwan. This comprehensive core set focuses on (*a*) the four domains and nature of CC in AAC practice; (*b*) CAD aged 0 to 18 years who use AAC devices; (*c*) the availability of self-assessment, as well as the assessments of CPs and healthcare providers. Most importantly, this tool provides information regarding the follow-up benefits of subsidized AAC devices provided to CAD by the MOHW in Taiwan.

## 2. Methods

The present study was completed in 2019 and used two of the best-known consensus methods: the Delphi method and the nominal group technique (NGT), also known as the expert panel technique, to develop standards for the appropriateness of assessment in medical and health services research [54]. The Delphi method often pools and cultivates the options of a heterogeneous group to assess consensus about a given issue [54,55], and has been used to develop standards for the appropriateness of assessments in medical and health services research [54]. A correct response was not expected from the various rounds of the questionnaire. However, this technique avoids the dominance of a response from a single source, and shows summaries of distributed responses from the group [54]. NGT is structured as a face-to-face expert panel within the context of a focus group for developing solutions (e.g., categorizations) [56].

### 2.1. Participants

A diverse combination of 14 Mandarin-speaking practitioners who are AAC experts, including five SLPs, five special education teachers, and four scholars signed informed consent forms and completed the entire research procedure [54]; the recommended minimum of seven participants for the Delphi method was met [57,58]. The participants were familiar with the framework and coding system of the ICF-CY and spoke Mandarin as their first language. The SLPs and special education teachers have worked for at least five years in clinical practice and special education, respectively, and have at least one year of experience providing AAC services in Taiwan (e.g., assessment and/or intervention) to Mandarin-speaking CAD. The scholars had to have earned their master’s or doctoral degree in communication disorders or special education, and conducted AAC-related research on Mandarin-speaking CAD with complex communication needs. Table 1 outlines the participants’ demographic information.

### 2.2. Procedures and Data Analysis

Second-level (i.e., 3-digit) ICF-CY codes are recommended for surveys and the evaluation of clinical outcomes [31]. The current study aimed to develop a core set using the full range of second-level codes [59]. The first step entailed using the Delphi method to identify codes relevant to the four domains of CC, and the second step required using the NGT to categorize the selected codes into one of the four domains. The first step was performed using the Delphi method to reach a consensus among the participants regarding the selected codes [60,61]; none of the participants were aware of the other participants [62]. The second-level codes were not pre-chosen to avoid bias [40]. A three-round Delphi method was conducted through an online, close-ended questionnaire (i.e., Google Docs) provided in Mandarin [60,61]. The questionnaire was sent out by e-mail to all participants and contained: (*a*) the questionnaire’s purpose; (*b*) instructions; (*c*) a detailed timeline; and (*d*) demographic information (e.g., e-mail address, gender, age, role/profession, years of work experience, and years of providing AAC services); (*e*) definitions of the four domains of CC from the work of Light [10] and Light, Beukelman and Reichle [11]; and (*f*) the second-level codes [62]. The participants had four weeks to respond, and reminders were sent out approximately one week before the deadline.

The first round of the questionnaire asked the participants to review the definitions of the four domains of CC. Next, they were asked to consider whether each second-level ICF code was relevant to any domain of CC. To turn the codes into a core set, the participants were provided with the following directions as suggested by Rowland, Fried-Oken, Lollar, Phelps, Simeonsson and Granlund [28]:

According to the definitions of the four domains of [CC] proposed by Light [10] and Light, Beukelman and Reichle [11], please rate the potential relatedness of each [ICF-CY] code to each domain of CC on a 5-point Likert scale, including (1) the item is not relevant; (2) the item is relevant; (3) the item is relevant and [we should be concerned about it]; (4) uncertain (considered relevant); and (5) uncertain (considered not relevant).

After the first round, the relatedness of each code was analyzed for selection and elimination. Responses of (2) the item is relevant; (3) the item is relevant and [we should be concerned about it]; and (4) uncertain (considered relevant) were grouped under the “relevant group,” while responses to (1) the item is not relevant, and (5) uncertain (considered not relevant) were placed in the “irrelevant group.” Descriptive statistics were used to examine the frequency with which the participants rated the potential relatedness of each ICF-CY code. Codes that received less than 40% of all participant ratings in the “irrelevant group” were automatically removed, while codes that received at least 75% of all participant ratings in the “relevant group” were automatically included [49,63]. Codes that received 40 to 74% relevance from all participants were deemed ambiguous and listed in an upcoming round of the questionnaire [49].

The questionnaire of the second round was similar to the one carried out in the first round but included: (*a*) a summary list of all ambiguous codes and (*b*) percentages of the participants who had considered the ambiguous codes relevant to CC [49,62]. The participants were asked to consider whether each of the second-level ICF-CY codes was relevant to any domain of CC. Similar descriptive statistics were conducted.

The third round of the Delphi method was performed in the same way as the second round; the participants were also asked to consider whether each of the second-level ICF-CY codes was relevant to any aspect of CC. The Delphi method was discontinued when all codes were automatically included (i.e., codes rated as the “relevant group” by at least 75% of all participants) or removed (i.e., codes rated as the “irrelevant group” by less than 40% of all participants). Conversely, it continued to be utilized when the codes received 40 to 74% relevance from all participants [49]. Three rounds of the Delphi method were carried out for the current study.

In the second step, each selected code from the Delphi method identified in the first step was placed under one of the four domains of CC. The NGT (involving a face-to-face expert panel), performed in Mandarin, was used to reach a consensus on the categorization of the codes. The written definitions of the four domains of CC proposed by Light [10] and Light, Beukelman and Reichle [11] were provided for discussion. Next, each participant independently categorized each item. Two rounds of the NGT were carried out, similar to the Delphi method, and the percentages of the categorizations of each code into the five groups were tallied. Each participant was provided with the following instructions:

According to the definitions of the four domains of CC proposed by Light [10] and Light, Beukelman and Reichle [11], please categorize each item into one of the following five groups, including (1) linguistic competence; (2) operational competence; (3) social competence; (4) strategic competence; and (5) none of these.

## 3. Results

Three rounds of the Delphi method were performed through the online closed-ended questionnaire to reach a consensus on the related codes from the participants, and the NGT was employed to categorize each selected code into one of the four domains of CC. The second-level codes listed in the ICF-CY were initially presented. After the first round of the Delphi method, 117 codes—48 (41%) from *Body Functions (b)*, 6 (5%) from *Body Structures (s)*, 51 (44%) from *Activities and Participation (d)*, and 12 (10%) from *Environmental Factors (e)*—were included. Five codes receiving 40 to 74% relevance from all participants were considered ambiguous and listed in the second round of the questionnaire. After the second round of the Delphi method was carried out, 114 codes—47 (41%) from *Body Functions (b)*, 6 (5%) from *Body Structures (s)*, 50 (44%) from *Activities and Participation (d)*, and 11 (10%) from *Environmental Factors (e)*—were included. Two codes receiving 40 to 74% relevance from all participants were considered ambiguous and listed in the second round of the questionnaire. After the third and final round of the Delphi method was performed, 112 codes—47 (42%) from *Body Functions (b)*, 6 (5%) from *Body Structures (s)*, 50 (45%) from *Activities and Participation (d)*, and 9 (8%) from *Environmental Factors (e)*—were included. Appendix A contains the 112 s-level codes in the core set, with their components and chapters listed in the ICF-CY.

These 112 codes were fully categorized into the four domains of CC through the NGT, as reported in Appendix A. None of them were categorized under (5) none of these. Twenty-eight codes were further classified under linguistic competence, 38 under operational competence, 32 under social competence, and 20 under strategic competence. Among these, “*b125 dispositions and intra-personal functions*”, “*b140 attention functions*”, “*b164 higher-level cognitive functions*”, “*d155 acquiring skills*”, and “*d230 carrying out daily routine*” were placed under social and strategic competence. The code “*b130 energy and drive functions*” was classified under operational and social competence. The code “*b163 basic cognitive functions*” was placed under linguistic and strategic competence.

## 4. Discussion

The ICF-CY component *Activities and Participation (d)* contained the largest number of codes, followed by *Body Functions (b)*, *Environmental Factors (e)*, and *Body Structures (s)*, respectively. There are several possible explanations for these outcomes. First, the components of *Body Functions (b)*, *Activities and Participation (d)*, and *Environmental Factors (e)* are the areas that SLPs and special education teachers mainly work on during clinical practice and special education, respectively, compared to *Body Structures (s)* [28]. Second, several codes in *Activities and Participation (d)* and *Environmental Factors (e)* link communication well, and soundly describe CC [24,36,41]. The codes in *Environmental Factors (e)* refer to the supports and barriers of *Environmental Factors (e)*, such as socio-relational skills in social competence. Third, barriers to participation, as considered in the participation model in AAC practice, can be comprehensively reflected in CC [17,52]. The codes in *Body Functions (b)*, *Activities and Participation (d)*, and *Environmental Factors (e)* play a more critical role than those of *Body Structures (s)* in this ICF-CY core set developed for measuring CC in dyadic communication with CAD who use AAC devices.

The codes in the core set were fully categorized into the four domains of CC (i.e., linguistic, operational, social, and strategic). Operational competence (38 codes) encompasses the largest number of codes, followed by social competence (33 codes) and linguistic competence (27 codes). Strategic competence (21 codes) contains the smallest number of codes. This is a logical outcome because the four domains of CC are interrelated [8] and the first three competencies develop simultaneously, while strategic competence emerges later [15]. Further, the small number of codes in strategic competence might be explained by the fact that adaptive skills are learned and used later than other competencies to minimize and compensate for restrictions in linguistic, operational, and/or social competence [14,64]. Different CPs (e.g., immediate and extended family members) use diverse adaptive skills to communicate with CAD who use AAC to co-construct their CC [11,16,23].

Several codes were simultaneously placed under two domains. This finding is corroborated by Blischak, Loncke and Waller [15], who confirmed that more than one CC domain might be concurrently involved in communication. First, the codes *b125, b140, b164, d155*, and *d230* were classified under social and strategic competence. In social competence, *b125* illustrates personal communicative confidence in initiating, maintaining, developing, and terminating communication to attain social closeness with diverse CPs. The appropriate adaptive skills selected by CAD according to varied CPs and environments (strategic competence) are also reflected in *b125*. Social competence in making decisions on how to initiate, maintain, and terminate communication, judge communication breakdowns, and then meta-cognitively plan to select appropriate adaptive skills in strategic competence is exemplified in *b140* and *b164*. The learned skills in sequentially initiating, maintaining, and terminating communication (social competence), and planning adaptive skills that match communication breakdowns according to varied environments and CPs (strategic competence) are exemplified in *d155* and *d230*.

Second, *b130* was categorized into operational and social competence; *b130* reflects mental functions of physiological (e.g., cognition) and psychological (e.g., motivation) mechanisms that enable people to satisfy their individual needs and goals [31]. Motivation (e.g., trusting AAC systems and devices) and cognition (e.g., understanding the required motor planning steps)—which are needed for operational competence—are reflected in *b130*. Social communication skills to meet communication goals in social competence are contained in *b130* as well. Third, *b163* was placed under both linguistic and strategic competence and reveals the ability to comprehend and express speech and AAC symbols, as well as to organize and apply them in communication.

## 5. Limitations

Although the ICF-CY core set was decided upon through two steps (the Delphi method and the NGT), there are still some limitations. First, the participant sample (i.e., 14) might not be representative of the larger population of practitioners who are AAC experts. In addition, the consensus of the recruited participants might differ from that of other observers, including CAD who use AAC devices, parents, or healthcare providers (e.g., the MOHW). Powell [65] stressed that the Delphi method is represented by the qualities—not the number—of participants. Linstone [57] further stated that a suitable minimum sample size is seven. A small number of participants in qualitative research typically produces a large amount of information [66]. Second, using the definitions of the four domains of CC proposed by Light [10] and Light, Arnold and Clark [13] in the Delphi method may have excluded some other appropriate definitions. However, several scholars [8,16,17,67] have argued that the definitions proposed by Light [10] and Light, Arnold and Clark [13] have dominated AAC research and practice for many years. Third, the ICF-CY core set was solely decided upon using the Delphi method, and the selected codes were placed into one of the four domains of CC through the NGT. Also, this tool was not validated, so the results should be interpreted with caution.

## 6. Implications

The assessment tool developed in this study for measuring CC by using the ICF-CY codes and guidelines for coding, acknowledged by the World Health Organization [WHO] [31,32], has several implications for clinical practice, despite the merger of the ICF and ICF-CY. First, the follow-up benefits of using the AAC devices subsidized by the MOHW among CAD and their CPs in daily communication life can now be assessed through the ICF and ICF-CY universal qualifiers (e.g., (4) complete impairment, difficulty or barrier), and, consequently, these subsidized AAC devices might not be abandoned. Second, the limitations and restrictions of the four domains of CC in dyadic communication can also be identified through the aforementioned universal qualifiers. Required eligibility for receiving and transitioning to social and educational services can be gauged [28,41]. Third, CC in dyadic communication among CAD who use AAC devices, and their peers in educational services, can be measured by practitioners across disciplines (e.g., special education teachers) while considering environmental factors in [48]. Consequently, the limitations, restrictions, and barriers of CC can be highlighted and overcome [68,69,70].

## 7. Conclusions

The comprehensive codes of the developed ICF-CY core set corresponded more frequently to *Body Functions (b)* and *Activities and Participation (d)* compared to the other two components, and were most frequently placed under operational, social, and linguistic competence. The follow-up benefits of the AAC devices subsidized by the MOHW can be measured through this ICF-CY core set regarding CC in dyadic communication among CAD who use AAC devices in daily life. In addition, the five levels of universal qualifiers (e.g., (4) complete impairment, difficulty or barrier) that establish the relative levels of CC across diverse communication contexts (e.g., discussing food) and dyads (e.g., peers, family members) can be used in educational services for support and transitions. This core set can be rated by the primary communicators (e.g., CAD who use AAC devices), their CPs (e.g., family members who communicate with them), and healthcare providers (e.g., SLPs and special education teachers). However, further studies are needed to validate this ICF-CY core set in social services (i.e., AAC devices subsidized by the MOHW) and educational services (e.g., special education).

## Figures and Tables

**Table 1:** Participant Demographic Information.

No.	Professional Category	Gender	Age	Practice of Service (Years)	AAC Related Years of Service
1	Special Educator	Male	46	17	17
2	Special Educator	Male	32	6	6
3	Special Educator	Male	40	11	11
4	Special Educator	Female	36	11	11
5	Special Educator	Male	36	12	5
6	SLP	Male	40	15	15
7	SLP	Female	26	5	3
8	SLP	Female	38	10	3
9	SLP	Female	55	25	2.5
10	SLP	Female	48	24	19
11	Scholar	Female	50	20	10
12	Scholar	Female	36	15	15
13	Scholar	Male	53	25	12
14	Scholar	Female	35	13	13

## Data Availability

The data presented in this study are available in Table 1, Supplementary Materials and Appendix A.

## Supplementary Materials

The following supporting information can be downloaded at: https://www.mdpi.com/article/10.3390/bs12110467/s1. References [71,72,73] are cited in the Supplementary Materials.

## Appendix A

**Table A1:** Codes Listed in the ICF-CY for Profiling CC Categorized into the Four Domains.

Component	ICF-CY Code	Domains of CC
*Body Functions (b)*	b114 Orientation Functions	Social competence
	b117 Intellectual Functions	Linguistic competence
	b122 Global Psychosocial Functions	Social competence
	b125 Dispositions and Intra-Personal Functions	Social competence; Strategic competence
	b126 Temperament and Personality Functions	Social competence
	b130 Energy and Drive Functions	Operational competence; Social competence
	b140 Attention Functions	Social competence; Strategic competence
	b144 Memory Functions	Linguistic competence
	b147 Psychomotor Functions	Operational competence
	b152 Emotional Functions	Social competence
	b156 Perceptual Functions	Operational competence
	b160 Thought Functions	Linguistic competence
	b163 Basic Cognitive Functions	Linguistic competence
	b164 Higher-Level Cognitive Functions	Social competence; Strategic competence
	b167 Mental Functions of Language	Linguistic competence
	b176 Mental Function of Sequencing Complex Movements	Operational competence
	b180 Experience of Self and Time Functions	Social competence
	b210 Seeing Functions	Operational competence
	b215 Functions of Structures Adjoining The Eye	Operational competence
	b230 Hearing Functions	Operational competence
	b260 Proprioceptive Function	Operational competence
	b265 Touch Function	Operational competence
	b270 Sensory Functions Related to Temperature and Other Stimuli	Operational competence
	b310 Voice Function	Linguistic competence
	b320 Articulation Functions	Linguistic competence
	b330 Fluency and Rhythm of Speech Functions	Linguistic competence
	b340 Alternative Vocalization Functions	Linguistic competence
	b398 Voice and Speech Functions, Other Specified	Linguistic competence
	b399 Voice and Speech Functions, Unspecified	Linguistic competence
	b455 Exercise Tolerance Functions	Operational competence
	b710 Mobility of Joint Functions	Operational competence
	b715 Stability of Joint Functions	Operational competence
	b720 Mobility of Bone Functions	Operational competence
	b729 Functions of The Joints and Bones, Other Specified and Unspecified	Operational competence
	b730 Muscle Power Functions	Operational competence
	b735 Muscle Tone Functions	Operational competence
	b740 Muscle Endurance Functions	Operational competence
	b749 Muscle Functions, Other Specified and Unspecified	Operational competence
	b750 Motor Reflex Functions	Operational competence
	b755 Involuntary Movement Reaction Functions	Operational competence
	b760 Control Of Voluntary Movement Functions	Operational competence
	b761 Spontaneous Movements	Operational competence
	b765 Involuntary Movement Functions	Operational competence
	b780 Sensations Related To Muscles and Movement Functions	Operational competence
	b789 Movement Functions, Other Specified and Unspecified	Operational competence
	b798 Neuro-musculoskeletal and Movement-Related Functions, Other Specified	Operational competence
	b799 Neuro-musculoskeletal and movement-related functions, unspecified	Operational competence
*Body Structures (s)*	s320 Structure of Mouth	Linguistic competence
	s330 Structure of Pharynx	Linguistic competence
	s340 Structure of Larynx	Linguistic competence
	s398 Structures Involved in Voice and Speech, Other Specified	Linguistic competence
	s399 Structures Involved in Voice and Speech, Unspecified	Linguistic competence
	s730 structure of upper extremity	Operational competence
*Activities and Participation (d)*	d115 Listening	Linguistic competence
	d120 Other Purposeful Sensing	Operational competence
	d131 Learning Through Actions With Objects	Operational competence
	d132 Acquiring Information	Linguistic competence
	d135 Rehearsing	Linguistic competence
	d137 Acquiring Concepts	Linguistic competence
	d145 Learning to Write	Linguistic competence
	d150 Learning to Calculate	Linguistic competence
	d155 Acquiring Skills	Social competence; Strategic competence
	d160 Focusing Attention	Social competence
	d163 Thinking	Social competence
	d166 Reading	Linguistic competence
	d170 Writing	Linguistic competence
	d175 Solving Problems	Strategic competence
	d177 Making Decisions	Strategic competence
	d210 Undertaking A Single Task	Strategic competence
	d220 Undertaking Multiple Tasks	Strategic competence
	d230 Carrying Out Daily Routine	Social competence; Strategic competence
	d240 Handling Stress and Other Psychological Demands	Social competence
	d250 Managing One’s Own Behaviour	Social competence
	d310 Communicating With—Receiving—Spoken Messages	Linguistic competence
	d315 Communicating With—Receiving—Nonverbal Messages	Linguistic competence
	d332 Singing	Linguistic competence
	d335 Producing Nonverbal Messages	Linguistic competence
	d350 Conversation	Social competence
	d355 Discussion	Social competence
	d360 Using Communication Devices and Techniques	Strategic competence
	d369 Conversation and Use of Communication Devices and Techniques, Other Specified and Unspecified	Operational competence
	d399 Communication, Unspecified	Strategic competence
	d415 Maintaining a Body Position	Operational competence
	d429 Changing and Maintaining Body Position, Other Specified and Unspecified	Operational competence
	d430 Lifting and Carrying Objects	Operational competence
	d440 Find Hand Use	Operational competence
	d445 Hand and Arm Use	Operational competence
	d446 Fine Foot Use	Operational competence
	d660 Assisting Others	Social competence
	d710 Basic Interpersonal Interactions	Social competence
	d720 Complex Interpersonal Interactions	Social competence
	d729 General Interpersonal Interactions, Other Specified and Unspecified	Social competence
	d730 Relating With Strangers	Social competence
	d740 Formal Relationships	Social competence
	d750 Informal Social Relationships	Social competence
	d760 Family Relationships	Social competence
	d770 Intimate Relationships	Social competence
	d779 Particular Interpersonal Relationships, other Specified and Unspecified	Social competence
	d798 Interpersonal Interactions and Relationships, other Specified	Social competence
	d799 Interpersonal Interactions and Relationships, Unspecified	Social competence
	d880 Engagement in Play	Social competence
	d910 Community Life	Social competence
	d920 Recreation and Leisure	Social competence
*Environmental Factors (e)*	e125 Products and Technology for Communication	Strategic competence
	e130 Products and Technology for Education	Strategic competence
	e310 Immediate Family	Strategic competence
	e315 Extended Family	Strategic competence
	e320 Friends	Strategic competence
	e325 Acquaintances, Peers Colleagues, Neighbors and Community Members	Strategic competence
	e340 Personal Care Providers and Personal Assistants	Strategic competence
	e415 Individual Attitudes of Extended Family Members	Strategic competence
	e440 Individual Attitudes of Personal Care Providers and Personal Assistants	Strategic competence

## References

[1] Department of Statistics (2022). Monthly Bulletin of Interior Statistics.

[2] Ministry of Health and Welfare [MOHW] (2022). Number of Children with Disabilities by Type of Disability.

[3] Beukelman D.R., Mirenda P. (2020). Augmentative and Alternative Communication: Supporting Children and Adults with Complex Communication Needs.

[4] Ministry of Health and Welfare [MOHW] (2012). Regulations on Selection and Training of the Professional Workers Providing Welfare Service for the Disabled.

[5] Social and Family Affairs Administration [SFAA] Assistive Device Evaluation Report 12. https://www.sfaa.gov.tw/SFAA/Pages/ashx/File.ashx?FilePath=~/File/Attach/7536/File_172882.pdf.

[6] Tsai M.-J. (2022). Development of ICF core set to profile communicative competence in dyadic communication among adults who use communication devices in Taiwan. Disabil. Rehabil. Assist. Technol..

[7] Light J.C., McNaughton D. (2014). Communicative competence for individuals who require augmentative and alternative communication: A new definition for a new era of communication?. Augment. Altern. Commun..

[8] Light J.C., Light J.C., Beukelman D.R., Reichle J. (2003). Shattering the silence: Development of communicative competence by individuals who use AAC. Communicative Competence for Individuals Who Use AAC.

[9] Gertner B.L., Rice M.L., Hadley P.A. (1994). Influence of communicative competence on peer preferences in a preschool classroom. J. Speech Hear. Res..

[10] Light J.C. (1989). Toward a definition of communicative competence for individuals using augmentative and alternative communication systems. Augment. Altern. Commun..

[11] Light J.C., Beukelman D.R., Reichle J. (2003). Communicative Competencies for Individuals Who Use AAC: From Research to Effective Practice.

[12] Bailey R.L., Stoner J.B., Parette H.P., Angell M.E. (2006). AAC team perceptions: Augmentative and alternative communication device use. Educ. Train. Dev. Disabil..

[13] Light J.C., Arnold K.B., Clark E.A., Light J.C., Beukelman D.R., Reichle J. (2003). Finding a place in the “social circle of life”. Communicative Competence for Individuals Who Use AAC: From Research to Effective Practice.

[14] Savignon S.J. (1983). Communicative Competence: Theory and Classroom Practice. Texts and Contexts in Second Language Learning.

[15] Blischak D.M., Loncke F., Waller A., Lloyd L.L., Fuller D.R., Arvidson H.H. (1997). Intervention for persons with developmental disabilities. Augmentative and Alternative Communication: A Handbook of Principles and Practices.

[16] Tsai M.-J. (2016). Revisiting communicative competence in augmentative and alternative communication. Folia Phoniatr. Logop..

[17] Teachman G., Gibson B.E. (2014). ‘Communicative competence’ in the field of augmentative and alternative communication: A review and critique. Int. J. Lang. Commun. Disord..

[18] Tsai M.-J. (2013). Rethinking communicative competence for typical speakers: An integrated approach to its nature and assessment. Pragmat. Cogn..

[19] Tsai M.-J., Chih Y.-C. (2021). Conversation turns and speaking roles contributed by Mandarin Chinese dyadic conversations between adults who use speech-generating devices and adults who use natural speech. Assist. Technol..

[20] Blackstone S.W., Berg M.H., Wilkins D.P. Social networks & AAC: Does what we do matter?. Proceedings of the 2005 American Speech-Language-Hearing Association Convention.

[21] Tsai M.-J., Scherz J., DiLollo A. (2011). Conversation of augmented and typical speakers—Speaking roles versus conversation turns. Asia Pac. J. Speech Lang. Hear..

[22] Tsai M.-J. (2013). The effect of familiarity of conversation partners on conversation turns contributed by augmented and typical speakers. Res. Dev. Disabil..

[23] Solomon-Rice P., Soto G. (2011). Co-construction as a facilitative factor in supporting the personal narratives of children who use augmentative and alternative communication. Commun. Disord. Q..

[24] O’Halloran R., Larkins B. (2008). The ICF activities and participation related to speech-language pathology. Int. J. Speech-Lang. Pathol..

[25] Light J.C. (1988). Interaction involving individuals using augmentative and alternative communication systems: State of the art and future directions. Augment. Altern. Commun..

[26] Kangas K.A. (1991). Relationship of Communication Speed and Rate to the Perceived Communicative Competence of High School AAC Users. Doctoral Dissertation.

[27] Light J.C. (1996). Exemplary Practices to Develop the Communicative Competence of Students Who Use Augmentative and Alternative Communication.

[28] Rowland C., Fried-Oken M., Lollar D., Phelps R., Simeonsson R.J., Granlund M. (2012). Developing the ICF-CY for AAC profile and code set for children who rely on AAC. Augment. Altern. Commun..

[29] Rowland C., Fried-Oken M., Bowser G., Granlund M., Lollar D., Phelps R., Simeonsson R.J., Steiner S.A. (2016). The Communication Supports Inventory-Children & Youth (CSI-CY), a new instrument based on the ICF-CY. Disabil. Rehabil..

[30] Tobii Dynavox The Dynamic AAC Goals Grid 2. http://tdvox.web-downloads.s3.amazonaws.com/MyTobiiDynavox/dagg%202%20-%20writable.pdf.

[31] World Health Organization [WHO] (2007). International Classification of Functioning, Disability, and Health: Children & Youth Version: ICF-CY.

[32] World Health Organization [WHO] (2001). International Classification of Functioning, Disability and Health: ICF.

[33] Pless M., Granlund M. (2012). Implementation of the International Classification of Functioning, Disability and Health (ICF) and the ICF Children and Youth Version (ICF-CY) within the context of augmentative and alternative communication. Augment. Altern. Commun..

[34] Bernabeu M., Laxe S., Lopez R., Stucki G., Ward A., Barnes M., Kostanjsek N., Reed G., Tate R., Whyte J. (2009). Developing core sets for persons with traumatic brain injury based on the international classification of functioning, disability, and health. Neurorehabilit. Neural Repair.

[35] Wofford M.C., Ogletree B.T., Nardo T.D. (2022). Identity-focused practice in augmentative and alternative communication services: A framework to support the intersecting identities of individuals with severe disabilities. Am. J. Speech-Lang. Pathol..

[36] Simeonsson R.J., Björck-Åkessön E., Lollar D.J. (2012). Communication, disability, and the ICF-CY. Augment. Altern. Commun..

[37] Schiariti V., Masse L.C., Cieza A., Klassen A.F., Sauve K., Armstrong R., O’Donnell M. (2014). Toward the development of the international classification of functioning core sets for children with cerebral palsy: A global expert survey. J. Child Neurol..

[38] World Health Organization [WHO] Merging ICF-CY into ICF. https://www.who.int/docs/default-source/classification/icf/whoficresolution2012icfcy.pdf?sfvrsn=2c8e5e9b_4.

[39] McCormack J., McLeod S., Harrison L.J., McAllister L. (2010). The impact of speech impairment in early childhood: Investigating parents’ and speech-language pathologists’ perspectives using the ICF-CY. J. Commun. Disord..

[40] Spoorenberg L.W., Reijneveld S.A., Middel B., Uittenbroek R.J., Kremer P.H., Wynia K. (2015). The geriatric ICF core set reflecting health-related problems in community-living older adults aged 75 years and older without dementia: Development and validation. Disabil. Rehabil..

[41] Threats T.T., Worrall L. (2004). Classifying communication disability using the ICF. Adv. Speech Lang. Pathol..

[42] Raghavendra P., Bornman J., Granlund M., Björck-Åkesson E. (2007). The World Health Organization’s International Classification of Functioning, Disability and Health: Implications for clinical and research practice in the field of augmentative and alternative communication. Augment. Altern. Commun..

[43] Aiachini B., Cremascoli S., Escorpizo R., Pistarini C. (2016). Validation of the ICF core set for vocational rehabilitation from the perspective of patients with spinal cord injury using focus groups. Disabil. Rehabil..

[44] Simeonsson R.J., Sauer-Lee A., Granlund M., Björck-Åkesson E., Mpofu E., Oakland T. (2010). Developmental and health assessment in rehabilitation with the international classification of functioning, disability and health for children and youth. Rehabiltaion and Health Asesment: Aplying ICF Guidelines.

[45] Pan Y.-L., Hwang A.-W., Simeonsson R.J., Lu L., Liao H.-F. (2015). ICF-CY code set for infants with early delay and disabilities (EDD Code Set) for interdisciplinary assessment: A global experts survey. Disabil. Rehabil..

[46] Dreinhöfer K., Stucki G., Ewert T., Huber E., Ebenbichler G., Gutenbrunner C., Kostanjsek N., Cieza A. (2004). ICF core sets for osteoarthritis. J. Rehabil. Med..

[47] Khan F., Pallant J.F. (2007). Use of the International Classification of Functioning, Disability and Health (ICF) to identify preliminary comprehensive and brief core sets for multiple sclerosis. Disabil. Rehabil..

[48] Bileviciute-Ljungar I., Schult M., Borg K., Ekholm J. (2020). Preliminary ICF core set for patients with myalgic encephalomyelitis/chronic fatigue syndrome in rehabilitation medicine. J. Rehabil. Med..

[49] Selb M., Escorpizo R., Kostanjsek N., Stucki G., Üstün B., Cieza A. (2015). A guide on how to develop an international classification of functioning, disability and health core set. Eur. J. Phys. Rehabil. Med..

[50] Kaech Moll V.M., Escorpizo R., Portmann Bergamaschi R., Finger M.E. (2016). Validation of the comprehensive ICF core set for vocational rehabilitation from the perspective of physical therapists: International Delphi survey. Phys. Ther..

[51] Yen T.-H., Liou T.-H., Chang K.-H., Wu N.-N., Chou L.-C., Chen H.-C. (2014). Systematic review of ICF core set from 2001 to 2012. Disabil. Rehabil..

[52] Fried-Oken M., Granlund M. (2012). AAC and ICF: A good fit to emphasize outcomes. Augment. Altern. Commun..

[53] Topia M., Hocking C. (2012). Enabling development and participation through early provision of augmentative and alternative communication. N. Z. J. Occup. Ther..

[54] Jones J., Hunter D. (1995). Qualitative research: Consensus methods for medical and health services research. Br. Med. J..

[55] Turoff M., Linstone H.A. (2002). The Delphi Method: Techniques and Applications.

[56] Harvey N., Holmes C.A. (2012). Nominal group technique: An effective method for obtaining group consensus. Int. J. Nurs. Pract..

[57] Linstone H.A., Fowlers J. (1978). The Delphi Technique. Handbook of Futures Research.

[58] Thangaratinam S., Redman C.W. (2005). The delphi technique. Obstet. Gynaecol..

[59] Worrall L.E., Hickson L. (2008). The use of the ICF in speech-language pathology research: Towards a research agenda. Int. J. Speech-Lang. Pathol..

[60] Martino J.P. (1993). Technological Forecasting for Decision Making.

[61] Rowe G., Wright G., Bolger F. (1991). Delphi: A reevaluation of research and theory. Technol. Forecast. Soc. Chang..

[62] Weigl M., Cieza A., Andersen C., Kollerits B., Amann E., Stucki G. (2004). Identification of relevant ICF categories in patients with chronic health conditions: A Delphi exercise [Supplemental material]. J. Rehabil. Med..

[63] Grill E., Ewert T., Chatterji S., Kostanjsek N., Stucki G. (2005). ICF Core Sets development for the acute hospital and early post-acute rehabilitation facilities. Disabil. Rehabil..

[64] Hymes D.H. (1974). Foundations in Sociolinguistics: An Ethnographic Approach.

[65] Powell C. (2003). The Delphi technique: Myths and realities. J. Adv. Nurs..

[66] Curtis S., Gesler W., Smith G., Washburn S. (2000). Approaches to sampling and case selection in qualitative research: Examples in the geography of health. Soc. Sci. Med..

[67] Light J.C., Gulens M., Beukelman D., Yorkston K.M., Reichle J., Beukelman D.R., Reichle J. (2000). Rebuilding communicative competence and self-determination. Augmentative and Alternative Communication for Adults with Acquired Neurologic Disorders.

[68] Østvik J., Ytterhus B., Balandin S. (2017). Friendship between children using augmentative and alternative communication and peers: A systematic literature review. J. Intellect. Dev. Disabil..

[69] Østvik J., Balandin S., Ytterhus B. (2018). Interactional facilitators and barriers to social relationships between students who use AAC and fellow students. Soc. Health Vulnerability.

[70] Pennington L., Marshall J., Goldbart J. (2007). Describing participants in AAC research and their communicative environments: Guidelines for research and practice. Disabil. Rehabil..

[71] Hymes D.H., Bauman R., Sherzer J. (1974). Ways of speaking. Explorations in the Ethnography of Speaking.

[72] Rowland C. (2004). Communication Matrix.

[73] Bolton S.O., Dashiell S.E. (1984). INCH: Interaction Checklist for Augmentative Communication.

