# Supplementary material for: Using the ICF Framework to Assess Communicative Competence in Dyadic Communication among Children and Adolescents Who Use Augmentative and Alternative Communication Devices in Taiwan
Source: Behav Sci (Basel). 2022 Nov 21;12(11):467. doi: 10.3390/bs12110467 (PMC9687965; doi:10.3390/bs12110467)
Supplement: Supplementary file 1 [file behavsci-12-00467-s001.zip › behavsci-1992565-supplementary.pdf]

**Supplemental Materials**

The Communicative Competence Rating Scale (CCRS) was developed by Kangas [26] to profile all four competencies of 11–18-year-old children and adolescents using speech-generating devices (SGDs). It includes 20 assessing items, worded in pairs, including one in positive wording and the others in negative wording, with a 5-point Likert scale indicating the extent of agreement to disagreement by communication partners and third-party observers. Higher scores indicate better communication competence.

The Communicative Competence Scale (CCS) was developed by Light [27] to investigate the communicative competence of 12–20-year-old students who use AAC. The CCS was developed based on the constructs of communicative competence proposed by Hymes [71], and includes 25 assessing items with a 5-point Likert scale (i.e., strongly disagree, disagree, neither agree nor disagree, agree, and strongly agree), producing a maximum of 125 points in total [cf. 27]. These items mainly focus on whether there is successful or effective communication across several communication partners (e.g., a group of people, familiar adults, unfamiliar adults, and anyone else) in different environments (e.g., schools and communities) and are profiled by third-party observers.

Rowland et al. (2012) developed an AAC profile to describe the communication strengths and needs of 5–20-year-olds with complex communication needs and use AAC through the ICF-CY framework. Three phases were conducted, namely: (1) code selection and development, (2) alpha testing and revision, and (3) beta testing and revision. Nine pragmatic items in the Communication Matrix [72] and five communication device items in the Interaction Checklist for Augmentative Communication-Revised (INCH) [73] were selected for the profile to capture all of the communication features of children who use AAC. There were 126 items in total, including 21 items in participation restrictions, 69 items in communication restrictions, 8 items in Body Functions (b) impairments, and 28 items in Environmental Factors (e) in this AAC profile. Minor changes to the official ICY-CY language have been made to ensure more standard American English wordings, and practitioners should be trained to conduct this profile. The profile is completed by third-party observers. Rowland, Fried-Oken, Bowser, Granlund, Lollar, Phelps, Simeonsson and Steiner [29] further developed this AAC profile as the Communication Supports Inventory-Children & Youth (CSI-CY).

The Dynamic AAC Goals Grid 2 (DAGG-2) [30] was developed by Tobii Dynavox in conjunction with Dynamic Therapy Associates of Kennesaw. DAGG-2 is a checklist containing Ability Level Continuum, Linguistic Competency, Operational Competency, Social Competency, and Strategic Competency, used to determine a person's level of communication skills in AAC. The potential target skills, strengths, and levels of cueing (i.e., natural cue, indirect cue,

direct verbal cue, direct pointer cure, and physical assistance) are marked by third-party observers.
